# Attitudes and practices of primary care physicians in Ankara about food allergy and anaphylaxis

**DOI:** 10.1186/2045-7022-1-S1-P42

**Published:** 2011-08-12

**Authors:** Aysegul Akan, Ersoy Civelek, Mustafa Erkocoglu, Celal Ozcan, Dilek Azkur, Muge Toyran, Yasemin Gokce, Tali Ozdemir, Sedat Guler, Can Naci Kocabas

**Affiliations:** 1 Ankara Hematology Onkology Children’s Training and Research Hospital, Pediatric Allergy, Ankara, Turkey; 2 Local Health Managers’ Office of Ankara, Ankara, Turkey

## Background

Because of the rise in the frequency, food allergies (FA) and consequently anaphylaxis (A) are becoming public health problems. The conciousness about prevention, diagnosis and therapy has to be improved, especially among primary care physicians (PCP).

## Methods

The survey including questions and case examples about FA and was given to PCP during the 2 meetings held by Local Health Managers' Office of Ankara.

## Results

Median of the age of the participants and duration of theri practice were 42.4±6.5 and17.0±6.4 years. The 72.4% of participants pointed out that lower than 5% of their pediatric patients had food allergy. The 36.6% of the participants thought that FA could be completely cured with therapy, the 36.9% stated that they didn't refer the patients suspected for FA.The 73.4% defined asthma as a significant risk factor of A for the patient with FA. Egg white was the most common food allergen (85.8%) defined by PCP and teh second common food allergen was cow’s milk (67.5%). The 4.3% of the participants had ever given a prescription of epinephrine otoinjector, 83% of them defined they know poorly about the use of epinephrine otoinjector. The 50% of PCP defined epinephrine as the first drug in a case with anaphylaxis, 60% of this group pointed out to give subcutaneously. Fifty two percent of PCP indicated that the education for FA and A in medical school was deficient.

## Conclusions

PCP have some information about FA, but they heve to be educated about practical applications about the diagnosis and management of FA and A. Otherwise we could neither know the real extent end burden of FA and A nor form right practice.

